# Multimodality Imaging of the Neglected Valve: Role of Echocardiography, Cardiac Magnetic Resonance and Cardiac Computed Tomography in Pulmonary Stenosis and Regurgitation

**DOI:** 10.3390/jimaging8100278

**Published:** 2022-10-10

**Authors:** Pietro Costantini, Francesco Perone, Agnese Siani, Léon Groenhoff, Giuseppe Muscogiuri, Sandro Sironi, Paolo Marra, Serena Carriero, Anna Giulia Pavon, Marco Guglielmo

**Affiliations:** 1 Radiology Department, Ospedale Maggiore della Carità University Hospital, 28100 Novara, Italy; 2 Cardiac Rehabilitation Unit, Rehabilitation Clinic “Villa delle Magnolie”, 81020 Castel Morrone, Italy; 3 School of Medicine and Surgery, University of Milano-Bicocca, 20126 Milan, Italy; 4 Department of Radiology, Istituto Auxologico Italiano, IRCCS (Istituto di Ricovero e Cura a Carattere Scientifico), San Luca Hospital, 20149 Milan, Italy; 5 Department of Radiology, ASST Papa Giovanni XXIII Hospital, 24129 Bergamo, Italy; 6 Postgraduate School in Radiodiagnostics, Università degli Studi di Milano, 20133 Milan, Italy; 7 Cardiocentro Ticino Institute, Ente Ospedaliero Cantonale, 6900 Lugano, Switzerland; 8 Department of Cardiology, Division of Heart and Lungs, Utrecht University Medical Center, Utrecht University, 3584CX Utrecht, The Netherlands

**Keywords:** multimodality imaging, echocardiography, cardiac magnetic resonance, computed tomography, echocardiography, CMR, CT, pulmonary stenosis, pulmonary regurgitation, valvulopathy, pulmonary valve

## Abstract

The pulmonary valve (PV) is the least imaged among the heart valves. However, pulmonary regurgitation (PR) and pulmonary stenosis (PS) can occur in a variety of patients ranging from fetuses, newborns (e.g., tetralogy of Fallot) to adults (e.g., endocarditis, carcinoid syndrome, complications of operated tetralogy of Fallot). Due to their complexity, PR and PS are studied using multimodality imaging to assess their mechanism, severity, and hemodynamic consequences. Multimodality imaging is crucial to plan the correct management and to follow up patients with pulmonary valvulopathy. Echocardiography remains the first line methodology to assess patients with PR and PS, but the information obtained with this technique are often integrated with cardiac magnetic resonance (CMR) and computed tomography (CT). This state-of-the-art review aims to provide an updated overview of the usefulness, strengths, and limits of multimodality imaging in patients with PR and PS.

## 1. Introduction

The pulmonary valve (PV) is located between the right ventricular outflow tract (RVOT) and the pulmonary artery (PA), inserted on a crown-like annulus. The valve consists of 3 cusps and commissures, similar to the aortic valve in anatomy but with thinner leaflets [1]. Specifically, two semilunar leaflets are posterior, named left and right, close to the left coronary cusp and right coronary cusp of the aortic valve, respectively. The other leaflet is positioned in front of the two posterior cusps and is named the anterior cusp. The three cusps move in the respective sinus of Valsalva, and with the fibrous interleaflet triangles and the distal part of right ventricle muscular infundibulum, constitute the pulmonary root complex [2].

Compared to other valvulopathies, PV diseases are less common and are typically associated with congenital heart disease.

The PV has a normal area of 2 cm^2^/m^2^. Pulmonary stenosis (PS) is a rare condition typically caused by congenital disease such as bicuspid, unicuspid, dysplastic valve, hypoplastic right heart ventricle, Ebstein anomaly or by acquired causes such as rheumatic disease, carcinoid disease, or tumors. Furthermore, PS is associated with complex congenital diseases such as univentricular heart, tetralogy of Fallot (TOF), and double outlet right ventricle (DORV) [3].

On top of the narrowing of the PV, subvalvular or supravalvular defects may also act with PS. Subvalvular stenosis is associated with TOF, ventricular septal defect, or is caused by hypertrophic cardiomyopathy, infiltrative cardiomyopathies, previous surgery on RVOT, and tumors. Supravalvular stenosis is caused by the narrowing of the PA [4].

PS causes pressure overload of the right ventricle (RV). Pressure overload increases wall stress, with an increase in contractility and a compensatory right ventricular hypertrophy to maintain a normal cardiac output. Right ventricular hypertrophy may cause a decrease in ventricular compliance, resulting in increased right ventricular end-diastolic pressures and increased right atrial pressures. Over time, progressive right ventricular hypertrophy and stiffness can determine right ventricular diastolic and systolic dysfunction [5].

Intervention should be considered in patients with severe symptomatic PV stenosis (peak gradient > 64 mmHg), but also in non-severe stenosis (peak gradient < 64 mmHg) in the presence of symptoms. This includes worsening right ventricular dysfunction and/or tricuspid regurgitation, concomitant atrial septal defect or ventricular septal defect with right-to-left shunt. Intervention with a catheter-based balloon valvotomy is recommended for PV stenosis and pulmonary arterial stenosis, while surgery is recommended in all other types of PS [6]. The indication for intervention in severe symptomatic RVOT obstruction is recommended in the case of surgical valve replacement.

Pulmonary regurgitation (PR) is quite a common finding during routinary echocardiography, and typically presents a good prognosis. However, it may be caused by congenital morphologic diseases, prolapse leaflets, post-repair of TOF, endocarditis, carcinoid syndrome, and rheumatic disease (primary PR), or it may be related to elevated PA pressure and dilated PA (secondary PR) [7].

Chronic severe PR may lead to RV dilation. Increased right ventricular end-diastolic volume allows for a compensatory increase in stroke volume to maintain cardiac output. However, chronic RV volume overload results in RV diastolic dysfunction, with elevation in the right ventricular end-diastolic pressure. Moreover, dilation of the RV and tricuspid annulus forms the substrate for functional tricuspid regurgitation, which compounds the volume load [5].

It is worth mentioning that the most common cause of PR is present in patients following the repair of TOF. In TOF, the definitive surgical repair involves the patch closure of the ventricular septal defect and access to the RVOT to relieve the obstruction. More than one-third of patients undergo placement of a transannular RVOT patch made of pericardium, Dacron, or polytetrafluoroethylene. Transannular patching provides excellent relief of the RVOT obstruction, but it invariably causes pulmonary insufficiency, hypokinesis and aneurysm of the RVOT, as well as fibrosis [8]. As there is a close relationship between the degree of pulmonary insufficiency, the right ventricular diastolic dimensions, and stroke volume, it is very important to investigate the changes in the right heart chambers to decide the right surgical time.

In patients that have undergone TOF repair, the indication for pulmonary valve replacement is recommended if the patient is symptomatic, in the presence of ventricular tachyarrhythmias or if a right ventricular dysfunction become manifest. Furthermore, pulmonary valve replacement can be beneficial in subjects with ventricular dilation or dysfunction. Instead, the indication for valve replacement in isolated PR resulting from the repair of PS is recommended in moderate or severe symptomatic PR and dilated RV or with reduced function. In moderate or severe asymptomatic PR, replacement can be effective in the presence of progressive dilatation and/or dysfunction of the RV and/or reduced exercise tolerance [9].

Transcatheter pulmonary valve implantation is a non-surgical option in patients with PS or PR, also after previous cardiac intervention such as right ventricle–to–pulmonary artery conduit [10,11]. The main advantage of this approach is the less invasive nature compared to surgery. However, risks and possible complications are present such as device dislodgement, stent valve fracture, pulmonary artery obstruction or injury, coronary extrinsic compression, and infective endocarditis after implantation [12].

It appears clear that correctly grading PS and PR and its hemodynamic repercussion is of crucial importance for the patient’s management and for the correct timing of the intervention.

The PV can be defined as a “neglected valve” as it is the less imaged between the cardiac valves, even less that the tricuspid valve, which is often addressed as the “forgotten valve” [13].

The aim of this paper is to provide an updated review of the diagnostic role of transthoracic (TTE) and transesophageal (TEE) echocardiography, cardiac magnetic resonance (CMR) and computed tomography (CT) in assessing patients with PS and PR.

### 1.1. Pulmonary Valve Stenosis: Role of Echocardiography

The echocardiographic assessment of PS is the first-line approach to evaluate the severity, anatomy and site of stenosis, aetiology, associated lesions, and the impact on cardiac chambers [14,15]. An assessment with TTE is performed using a parasternal short axis view with a focus also on PA and its bifurcation, a parasternal long axis view with the direction towards the right shoulder, a modified apical five-chamber view, and the subcostal view.

**Figure 1:**
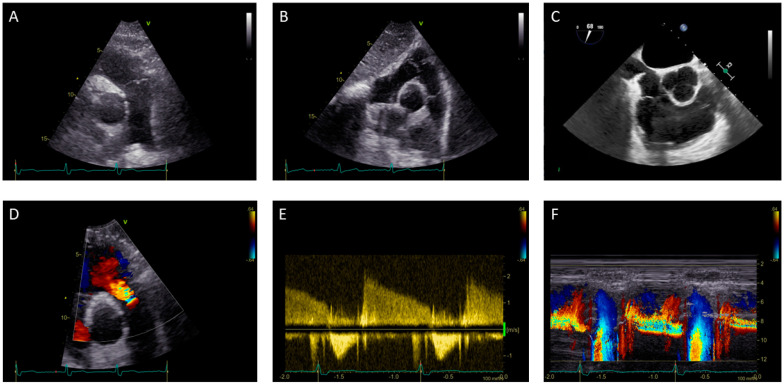
(**A**–**C**) Assessment with transthoracic and transesophageal echocardiography of the pulmonary valve in a 50-year-old female patient: (**A**) Parasternal short-axis view with focus on pulmonary valve and pulmonary artery; (**B**) Subcostal view; (**C**) Mid-esophageal right ventricle inflow-outflow view. (**D**–**F**) Transthoracic echocardiography assessment of pulmonary regurgitation in a 60-year-old male patient: (**D**) Detection of pulmonary regurgitation by color flow Doppler imaging; (**E**) Continuous wave Doppler of pulmonary regurgitation flow; (**F**) Color M-mode demonstrates holodiastolic regurgitation jet.

An assessment with TEE is performed during the mid-esophageal right ventricle inflow-outflow view, the mid and high-esophageal view at 90°, and the transgastric right ventricle inflow-outflow view [16] (Figure 1A–C).

Qualitative assessment is useful for assessing the stenosis site, the increased thickening and reduced mobility of the leaflets, calcification, and dome-shaped valve. Color-flow Doppler displays the aliasing level and localizes the stenosis.

PS grading is mainly based on the quantitative assessment of the pressure gradient (Table 1). Continuous-wave (CW) Doppler is aligned with the flow through the stenosis to obtain the velocity flow curve. At the same time, the peak velocity (m/sec) and the peak gradient (mmHg) are estimated. Mild PS is classified in the case of peak velocity < 3 m/sec and peak gradient < 36 mmHg, moderate PS in the case of peak velocity from 3 to 4/sec and peak gradient between 36 and 64 mmHg, and severe PS is indicated with peak velocity > 4 m/sec and peak gradient > 64 mmHg [4].

Valve orifice planimetry with a 2D approach is not recommended due to the significant limitation of obtaining the correct image plane. 3D planimetry could be performed but this approach is not validated [2]. Likewise, functional valve area calculation with continuity equation is infrequently performed due to the lack of validation and difficulty in measuring the RVOT.

The hemodynamic consequences of PS involve the right heart and PA, which can be identified with echocardiography. Parietal hypertrophy of the RV (RV free wall > 5 mm) can be measured in the subcostal view. Dilatation and dysfunction of the RV can be assessed as described in the Section 1.3.

Post stenotic pulmonary dilatation, right atrium dilatation, and high RV systolic pressure also are associated and can be identified with echocardiography.

In case of PS, pulmonary artery systolic pressure is calculated from the RV systolic pressure minus the PV pressure gradient. The RV systolic pressure is obtained from the tricuspid regurgitation velocity plus the right atrial pressure.

Finally, an echocardiography assessment is useful for evaluating the association of PS with other congenital lesions. Indeed, PS is associated with syndromes and congenital diseases such as univentricular heart, complete atrioventricular, TOF, and DORV [4].

### 1.2. Pulmonary Valve Regurgitation: Role of Echocardiography

The echocardiography assessment of PR is based on the integration of several indices such as qualitative, semi-quantitative, and quantitative parameters and the structural remodeling of the RV (Figure 1D–F). TTE is the first-line approach in PR assessment, while TEE has usually a limited role due to the distance of the probe from the valve [7].

Color flow imaging detects PR and defines a mild grade in the presence of a jet length < 10 mm with a narrow origin, while a severe regurgitation occurs in the case of a large jet width and wide origin. Instead, a ratio of jet width to PV annulus > 65% is indicative of severe PR [17]. Specifically, the maximum jet width is calculated just below the PV in diastole. Another parameter indicative of severe PR is the detection of diastolic reversal color flow in the PA branch. This index is more specific (87%), especially with holodiastolic regurgitation. In addition, it is suggested to sample the reverse flow in the PA with pulsed Doppler [18].

CW Doppler assessment adds significant features in defining severity [19]. Weak and slow deceleration of the signal is indicative of mild regurgitation, while dense and steep deceleration identifies as severe PR. In addition, the early termination of the diastolic regurgitant signal is compatible with severe PR. Pressure half-time measured in the parasternal short-axis view is associated with a severe PR with the value < 100 ms [20]. Furthermore, a PR index < 0.7 defines a severe regurgitation. This index is determined by the ratio of the PR duration to the total diastolic time [21].

Currently, key methods for defining other valvular regurgitant as vena contracta width and the proximal isovelocity surface area (PISA) method are not validated for PR, and the severity values are not defined.

A quantitative parameter for assessing the PR severity is the measurement of the regurgitant fraction using the pulsed Doppler methods. The regurgitant fraction is obtained from the ratio of the regurgitant volume to the RVOT stroke volume. This method is valid with multiple and eccentric jets, but is limited by errors in the measurement such as the RVOT diameter [22]. A value < 20% defines a mild PR, while between 20 and 40% a moderate PR, and a value > 40% is associated with a severe PR. (Table 2)

### 1.3. Right Ventricle Assessment in Pulmonary Stenosis and Regurgitation: Role of Echocardiography

An echocardiographic assessment of the RV size and function is part of the PR and PS evaluation. RV assessment by 2D-echocardiography should be routinely performed using multiple views. Quantification of basal, mid, and longitudinal diameter is obtained from the RV-focused apical four-chamber view. Instead, the RVOT proximal diameter is measured in the parasternal long- or short-axis view while the RVOT distal diameter is measured in the parasternal short-axis view. In the subcostal view, the RV wall thickness is measured with the zoom mode and the focus is on the mid-wall. The RV longitudinal systolic function is evaluated measuring the tricuspid annular plane systolic excursion (TAPSE, mm) by M-mode, and the peak systolic velocity of the tricuspid annulus (S’, cm/sec) by the pulsed-wave Doppler Tissue Imaging (DTI). The RV global systolic function is estimated by calculating the fractional area change (FAC, %) in the RV-focused apical four-chamber view as 100 × [(end-diastolic area/end-systolic area)/end-diastolic area]. However, the RVOT contribution is not included in this parameter [23]. Conversely, 3D echocardiography allows for the calculation of volumes and ejection fraction including all three RV components. Inflow, apical portion, and outflow tract are incorporated in the 3D assessment for analysis and calculation of the RV size and global systolic performance. Finally, the RV global longitudinal strain (GLS, %) is an additional parameter for evaluating the longitudinal myocardial function. Strain analysis should be performed in the RV-focused apical four-chamber view including either the RV free wall and interventricular septum, or the free wall segments only. This parameter is useful for diagnosing a subclinical RV dysfunction, with an additional prognostic value [24].

## 2. Role of Cardiac Magnetic Resonance in Pulmonary Stenosis and Regurgitation

CMR is a non-ionizing imaging modality that can be valuable in order to picture the PV and abnormalities of the PA, and of the RV in patients with PR and PS. CMR can have advantages in assessing the PV compared to echocardiography. First, while PV can be difficult to visualize on either TTE or TEE, it is easy to visualize in either en-face or RVOT views using Steady-State Free Precession (SSFP) imaging. Second, CMR is currently the gold standard for the assessment of RV volumes and function. Third, using contrast agents, CMR offers the possibility of assessing the pulmonary arteries tree. Fourth, either PS and PR can be quantified via “through-plane” phase-contrast (PC) imaging [25]. The PC technique uses sequences with and without encoding of the bloodstream, producing images in magnitude (qualitative assessment of the flow) and in phase (quantitative aspect such as direction and velocity).

In the last few years, four-dimensional (4D) flow CMR has emerged as a promising tool to assess patients with both PS and PR [26]. 4D flow CMR is a phase-contrast sequence with flow-encoding in all three spatial directions. It is resolved relative to all three dimensions of space and to the dimension of time along the cardiac cycle [27]. This imaging technique has been used mainly in the research field, but it will probably gain further consideration in the future. The main limiting factors of using CMR are the long scan time and the presence of incompatible metal objects and devices. Furthermore, the long acquisition time and the need of patient compliance limits its use in the pediatric population.

### 2.1. Pulmonary Stenosis: Role of CMR

The use of SSFP cine imaging with retrospective electrocardiography (ECG) gating in patients with PS allows for the assessment of the morphology and the dynamics of the PV, PA and RVOT. SSFP allows for the identification of the anatomical localization of the stenosis, and to visualize secondary abnormalities related to the pressure overload.

**Figure 2:**
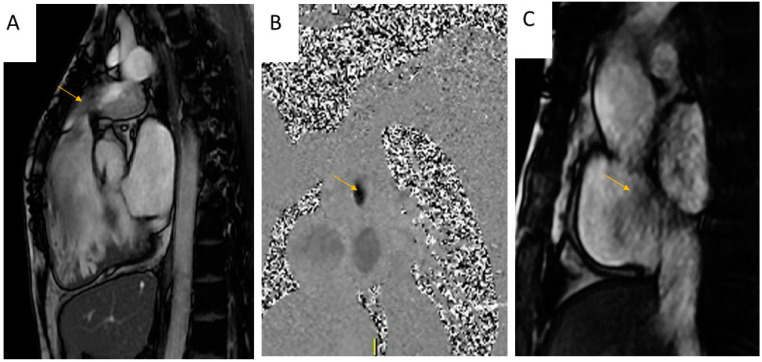
Assessment of Pulmonary Stenosis with Cardiac Magnetic Resonance: 2 (**A**,**B**) 40-year-old female patient with Pulmonary Stenosis. Visualization of a systolic “jet” (hypointense signal as a result of phase loss of proton spin due to turbulent flow) in pulmonary artery (arrow) with a cine-SSFP sequence. (**B**) Phase contrast sequence, visualization of hypointense signal at the level of luminal narrowing; 2 (**C**) 19-year-old male patient with pulmonary regurgitation, identification of diastolic “jet” in the right ventricle outflow tract.

CMR offers qualitative assessment of the regurgitation or stenosis with Steady-State Free Precession (SSFP) or Gradient Recalled Echo (GRE) RVOT [25]. SSFP and gradient echo cine images allow for the visualization of turbulent flow jets as signal voids (Figure 2A) [28].

Doppler echocardiography remains the most accurate, non-invasive imaging modality to grade PS. However, using phase-contrast sequences, peak velocities (correlated with the severity of the stenosis) can be precisely encoded at the level of luminal narrowing (Figure 2B). The appropriate change of the velocity encoding (VENC) is necessary to prevent aliasing [29].

PS is often common in TOF. The patient’s Magnetic Resonance Angiography (MRA) can help to identify the PA stenosis. Velocity mapping of the main pulmonary arteries may identify differences in flow between the stenotic and non-stenotic vessel. Due to its importance in TOF management and follow-up, and the difficulties in visualizing the PA via echocardiography, CMR should be performed at least once in these patients [30].

### 2.2. Pulmonary Regurgitation: Role of CMR

CMR is a valuable method to evaluate PR and the secondary RV remodeling [31]. Valve morphology (e.g., tricuspid, valvuloplasty for pulmonic stenosis) and pathology (e.g., endocarditis, carcinoid valve disease, rheumatic heart disease) as well as assessment of the RVOT and PA are crucial to picture the mechanisms of PR. Cine-SSFP imaging is the most used sequence for this purpose. Of note, CMR is not the best tool to assess the presence of small vegetations in IE, due to the non-real-time image acquisition over different cardiac cycles that can miss structures with asynchronous mobility [32].

As with PS, the turbulent flows created by the PR is represented as in the Cine-SSFP or GRE sequences as a “signal void” (Figure 2C).

Phase contrast velocity mapping through the gradient echo sequence can be performed above the PV to quantify the regurgitant flow (Figure 2A,B). According to the new consensus paper of the European association of cardiovascular imaging (EACVI), PR is defined as severe when the regurgitant fraction is >35% [33]. (Table 1)

### 2.3. Right Ventricle Assessment in Pulmonary Stenosis and Regurgitation: Role of Cardiac Magnetic Resonance

The anatomy and function of the RV with CMR can be obtained using balanced SSFP cine images in four-chamber, RV two-chamber and short axis views [34]. Another possible acquisition is performed via imaging in the axial plane of multiple axially rotated long-axis slices. Although this lengthens the exam duration, it offers a more precise evaluation of EF and RV in congenital heart diseases [24]. The RVOT view is obtained by aligning a plane through the main PA and the RV inferiorly from a set of axial images. Additionally, a perpendicular plane to the previous ones, in an axial or oblique axial orientation, can add more information about the RVOT [35].

The RV End Diastole Volume (EDV) and End Systole Volume (ESV) are measured manually by contouring the endocardium at the end-diastole and end-systole on SSFP cine loops, oriented along the short axis of the left ventricle or axial-oriented with a slice thickness of 4–8 mm [25]. EF is obtained by dividing the stroke volume (EDV–ESV) by EDV [30].

There is evidence that the remodeling process can be reversed by valve replacement until the CMR RV end-diastolic volume index is <163 mL/m^2^ or the end-systolic volume index is <80 mL/m^2^ [36]. In a study with 7131 patients with known or suspected cardiovascular disease, a RVEF calculated with CMR < 40% was linked to a 3.1-fold risk of major adverse cardiovascular events (MACE) [37].

Moreover, the RV global longitudinal strain (GLS) turned out to be a predictor of poor outcomes in operated TOF patients [38].

PR and PS are both associated with RV fibrosis [39] which can potentially cause arrhythmias. Due to the thin wall, late gadolinium enhancement (LGE) assessment in the RV is more problematic than in the left ventricle. However, there is evidence suggesting how LGE is also useful in patients with PR after TOF correction, and how the amount of fibrosis correlates with the severity of PR and RV dilatation [40,41]. Furthermore, RV diffuse fibrosis has been identified in corrected TOF using T1 mapping [42]. Secchi et al. demonstrated the negative correlation between the RV nT1 and the right ventricular ejection fraction (RVEF) [43].

## 3. Role of CT on Pulmonary Stenosis and Regurgitationt Assessment

CT is useful in obtaining precise anatomical information about the PV and contiguous anatomical structures, such as the RVOT the distal pulmonary arteries and coronary arteries [44]. To visualize the PV and the surrounding structures, it is necessary to adequately opacify the right heart chambers with an optimized imaging protocol.

A good attenuation of the right heart chambers can be obtained using a split-bolus injection, in which an initial bolus of 50–75 mL of contrast medium is followed by a bolus of 50 mL of saline and contrast medium (a mixture with a 70:30 ratio of saline to contrast medium) and a bolus of 30 mL of solution saline at a rate of 4–5 mL/s [45]. Dedicated CT of the right side of the heart requires ECG gating or triggering, and homogeneous enhancement of the right atrium and ventricle to an optimal level of 400–450 HU. Scanning may be started early (i.e., with main PA triggering) to include only the right side of the heart [8]. For morphological evaluation of the valve, prospective ECG-triggered acquisition is the default scanning mode to minimize the radiation dose. Nevertheless, if functional analysis of the valve or RV is desired, retrospective multiphasic acquisition with ECG with the tube current modulation is the ideal scanning mode [46]. With advances in CT scan technology, using multiplanar reformation (MPR) axial data can be reconstructed into sagittal and coronal images. Software analysis tools such as maximum intensity projection (MIP) and volumetric rendering (VR) techniques have improved diagnostic capabilities [47].

Accurate three-dimensional images obtained by CT scan help to assess the level of stenosis and the consequences of flow alterations.

Moreover, CT is useful for evaluation of both the native and prosthetic PV, and is the modality of choice for evaluation of the proximal and distal pulmonary arteries, including the coronary arterial anatomy [46,48]. The wide availability of CT and the rapid acquisition times make it a reasonable alternative to CMR, especially in small children and infants with complex cardiovascular malformations, despite considerations on the ionizing radiation dose.

### 3.1. Pulmonary Stenosis and CT

**Figure 3:**
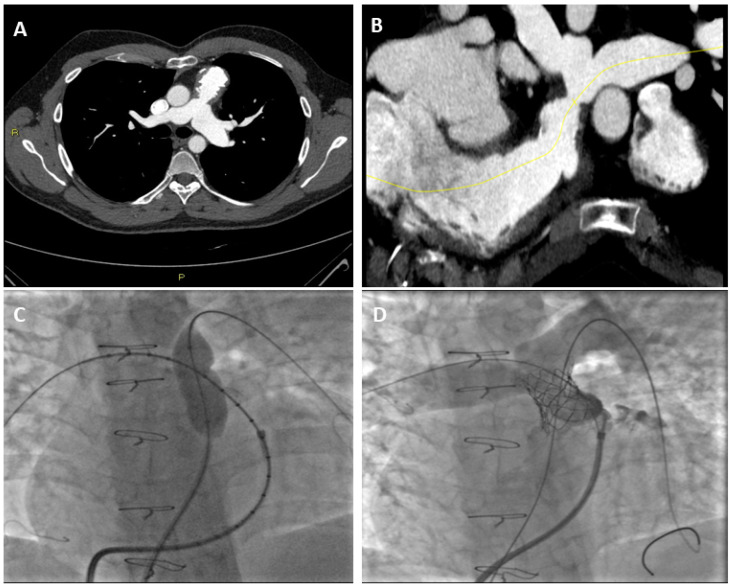
Assessment of Pulmonary Stenosis with CCT in a 15-year-old male patient: (**A**) Axial CT image with stenosis of the common trunk of the pulmonary artery; (**B**) CT reconstruction of the vessel using a dedicated post-processing software; (**C**) Angiographic image of percutaneous transluminal angioplasty (PTA) of the stenotic tract and (**D**) Angiographic image showing correct stent placement after PTA.

In patients with PS, CT angiography can assess the valvular morphology and the surrounding anatomy, and could also provide a functional assessment of the RV using post-processing software [49]. Compared with other diagnostic techniques, CT has a higher spatial resolution [50]. This allows for a more detailed assessment of the anatomy of small vessels and pulmonary veins. In patients with TOF, CT is important for planning surgical intervention. For example, the recognition of an abnormal coronary artery origin, such as the left anterior descending artery arising from the right coronary artery and passing through the RVOT, can preclude surgical procedures on the RVOT [51]. In adult patients, cardio CT is important in assessing the patency, integrity, morphology, and position of the stent (Figure 3). Finally, CT is used for the accurate measurement of the RVOT before transcatheter PV implantation [51].

### 3.2. Pulmonary Regurgitation and CT

Thanks to a detailed study of the anatomy of the valve and the surrounding structures, CT is useful to identify the cause of PR and for interventional planning. CT is the best modality for evaluating complications of endocarditis, especially in a patient with prosthetic valves, in which perivalvular infection is common. CT can help to identify valve dehiscence, transvalvular fistula, perivalvular abscess, and pseudoaneurysm [52]. Of note, as for CMR, CT sensitivity is reduced in the evaluation of small vegetations (<4 mm) which are best seen with echocardiography [53]. In carcinoid heart disease, generally both the tricuspid and pulmonary valves and their corresponding subvalvular apparatus are involved. CT can identify the thickening of the valve cusps, which become retracted and shortened, as well as the thickening and fusion of the chordae [8].

### 3.3. Right Ventricle Assessment with Cardiac CT

CT can be an alternative method to assess RV dimensions and EF in patients with contraindication to CMR. The volumes of the RV are measured with dedicated post-processing software on the RV short axis views using 10–20 phases of the cardiac cycle. EF is calculated using end-diastole (EDV) and end-systole volumes (ESV) [54]. The disadvantages of CT scanning are radiation doses and the use of contrast agents.

## 4. Prenatal Diagnosis of Pulmonary Stenosis and Regurgitation

Whenever possible, prenatal diagnosis of congenital heart disease should be obtained using echocardiography [55], considering the non-invasiveness and low cost. Vales et al. demonstrated that prenatal echocardiography could identify patients with pulmonary atresia and ventricular septal defect in 90% of patients [56].

However, sometimes prenatal diagnosis of congenital heart disease can be refined using CMR, especially in cases where the fetal echocardiography is limited by maternal obesity, oligohydramnios or the fetal position [57].

It is important to consider that the acquisition of CMR should be limited in specific cases, considering the potential fetal harm or hearing impairment. Despite this, the data is still debated in the literature [57].

Several techniques of CMR acquisition have been developed for the analysis of CHD. In particular, it is possible to acquire images without gating, or to use post-processing ECG gating with a time resolution of 12–80 ms [58]. Recently, Salehi et al. demonstrated the role of fetal CMR in assessing pulmonary atresia, allowing for the evaluation of intracardiac anatomy and biventricular function [59]. The possibility of increasing the diagnostic accuracy for the depiction of congenital heart disease was also confirmed by Zhen-Dong in a large cohort of patients where echocardiography was inconclusive [60]. The authors demonstrated that it was possible to identify patients with pulmonary atresia, with or without the associated ventricular septal defect. The correct assessment and confirmation of the pulmonary anomaly can be beneficial for planning surgical treatment.

## 5. Conclusions

Multimodality imaging offers several and complementary pieces of information in patients with pulmonary valvulopathy. Echocardiography represents the first step to assess patients with PS or PR in order to identify their mechanisms, to grade their severity and their hemodynamic consequences on RV such as parietal hypertrophy, dilatation and dysfunction. Echocardiography is often sufficient to diagnose the disease and for patient follow-up. Advanced imaging such as CMR and CT is needed, especially in suspected severe valvulopathies, when clinical data and echocardiographic findings are discordant and for a correct preoperative planning. CMR can be considered as a valuable additional modality in patients with PV disease. Not only is CMR the gold standard for measuring the volume of the RV and its function, but it also offers greater visualization of the PV and of the RVOT. Moreover, phase-contrast imaging can be used to assess the severity of both PS and PR. There is also some evidence that CMR identification of RV fibrosis with LGE and T1 mapping can be used to identify patients with a worse prognosis. CMR can be also used for prenatal diagnosis if echocardiography is inconclusive.

Finally, in patient candidates for surgery, thanks to its high spatial resolution, CT is the modality of choice to study the anatomy of the PV and the surrounding structures, including an accurate evaluation of the RVOT and pulmonary circulation. CT is also used to rule out coronary pathology and represents the best modality for evaluating complications, especially in patients with prosthetic valves.

## Figures and Tables

**Table 1:** Echocardiography grading of pulmonary stenosis (4).

PS Severity Classes	Mild	Moderate	Severe
Peak velocity (m/s)	<3	3–4	>4
Peak gradient (mmHg)	<36	36–64	>64

**Table 2:** Grading the severity of pulmonary regurgitation using echocardiography and cardiac magnetic (7).

*PR Severity Classes*	*Mild*	*Moderate*	*Severe*
**Echo Qualitative parameters**			
*Pulmonic valve morphology*	Normal	Normal/Abnormal	Abnormal
*Color flow PR jet width*	Small, usually <10 mm in length with a narrow origin	Intermediate	Large, with a wide origin; may be brief in duration
*Reversal flow in pulmonary artery branches*	Absent	Absent	**Present**
*CW signal of PR jet*	Faint/Slow deceleration	Dense/variable	Dense/steep deceleration, **early** **termination of diastolic flow**
*Pulmonic* vs. *aortic flow by PW*	Normal or slightly increased	Intermediate	Greatly increased
**Echo Semi-quantitative parameters**			
*VC width (mm)*	Not defined	Not defined	Not defined
*Deceleration time of the PR*	Not defined	Not defined	<260 ms
*Pressure half-time*	Not defined	Not defined	**<100 ms**
*Jet width/annulus ratio*	Not defined	Not defined	>65%
*PR index*	Not defined	Not defined	<0.77
**Echo Quantitative parameters**			
*EROA (mm^2^)*	Not defined	Not defined	Not defined
*R Vol (mL)*	Not defined	Not defined	Not defined
*RF (%)*	<20	20–40	>40
**CMR parameters**			
*RF (%)*	<20	20–40	>40
**Echo/CMR Structural parameters**			
*Structural parameters, RV size*	Usually normal	Normal or dilated	**Usually dilated**

Echo, echocardiography; CMR, cardiovascular magnetic resonance; CW, continuous wave; EROA, effective regurgitant orifice area; PR, pulmonic regurgitation; PW, pulse wave; RF, regurgitant fraction; R Vol, regurgitant volume; RV, right ventricle; VC, vena contracta. In bold: specific signs for severe PR.
